# A multi-species toolkit of *TOP2* hypercleavage mutants for studying topoisomerase II–mediated DNA damage

**DOI:** 10.64898/2026.05.09.723998

**Published:** 2026-08-11

**Authors:** David Ontoso, Monika Mehta, Aidin Shabro, John Dittmar, Robert J.D. Reid, Rodney Rothstein, John L. Nitiss, Scott Keeney

## Abstract

DNA topoisomerase II (TOP2) generates transient DNA double-strand breaks that are trapped as TOP2-DNA covalent complexes (TOP2cc) by antibiotic and chemotherapy drugs. Here, we characterize tools for study of cellular responses to TOP2cc, exploiting a *Saccharomyces cerevisiae TOP2* mutant ( *TOP2-F1025Y,R1128G* ) that generates spontaneous and inhibitor-induced covalent complexes at elevated frequencies. This Top2-hc (for “hypercleavage”) mutant protein inhibits yeast cell growth when expressed alone or with endogenous Top2, and growth defects are exacerbated in DNA-repair-deficient genetic backgrounds and/or in the presence of low doses of the Top2 poison mAMSA. We generated analogous mutations in human and mouse TOP2A and TOP2B that gave increased TOP2cc, hypersensitization to topoisomerase poisons, increased DNA damage, and decreased cell survival in cultured cells. We further established knock-in mouse models with inducible, tissue-specific expression of each TOP2-hc isoform, demonstrating overt organismal toxicity and cellular markers of DNA damage responses. To illustrate the potential of these genetic tools, we carried out proof-of-principle screens in yeast and cultured human cells for sensitivity to TOP2-hc. The yeast screen revealed strong requirements for homologous recombination, moderate roles for sister chromatid cohesion and kinetochore function, and dependencies on vesicle and vacuolar functions. The pilot shRNA screen in human cells revealed shared requirements for resistance to expression of either TOP2A-hc or TOP2B-hc as well as examples of isoform specificity. These findings establish hypercleavage mutant proteins as effective tools for studying topoisomerase isoform-specific DNA damage and offer a foundation for exploring TOP2cc toxicity and tolerance *in vivo* .

**Significance statement:**

DNA topoisomerase II enzymes untangle chromosomes by cutting DNA, but incomplete resealing creates toxic damage that is the basis of antibacterial and chemotherapy drugs. Here we describe toolkits in yeast, mammalian cells, and mice that take advantage of mutant topoisomerase II enzymes that trap on DNA without drugs, creating powerful genetic systems to better study how cells deal with this type of DNA damage. We provide benchmarking data to validate these tools and to illustrate how they can be used for screens in cultured cells or tissue-specific experiments *in vivo* . These toolkits overcome longstanding technical barriers and enable new ways to study topoisomerase II-mediated DNA damage.

## Full Text Availability

The license terms selected by the author(s) for this preprint version do not permit archiving in PMC. The full text is available from the preprint server.

